# Unexpected mosaic distribution of two hybridizing sibling lineages in the teleplanically dispersing snail *Stramonita haemastoma* suggests unusual postglacial redistribution or cryptic invasion

**DOI:** 10.1002/ece3.3418

**Published:** 2017-09-25

**Authors:** Tahani El Ayari, Najoua Trigui El Menif, Carlos Saavedra, David Cordero, Frédérique Viard, Nicolas Bierne

**Affiliations:** ^1^ Université de Montpellier Montpellier Cedex 5 France; ^2^ ISEM ‐ CNRS UMR 5554 Station Marine OREME Sète France; ^3^ Laboratory of Environment Bio‐monitoring Faculty of Sciences of Bizerta University of Carthage Bizerta Tunisia; ^4^ Instituto de Acuicultura Torre de la Sal Consejo Superior de Investigaciones Cientίficas Ribera de Cabanes (Castellόn) Spain; ^5^ UPMC Université Paris 6 CNRS UMR 7144 Adaptation et Diversité en Milieu Marin Equipe DIVCO Station Biologique de Roscoff Sorbonne Université Roscoff France

**Keywords:** biological invasion, cryptic species, hybrid zone, introgression, *Stramonita haemastoma*, Western Mediterranean Sea

## Abstract

Molecular approaches have proven efficient to identify cryptic lineages within single taxonomic entities. Sometimes these cryptic lineages maybe previously unreported or unknown invasive taxa. The genetic structure of the marine gastropod *Stramonita haemastoma* has been examined in the Western Mediterranean and North‐Eastern Atlantic populations with mtDNA *COI* sequences and three newly developed microsatellite markers. We identified two cryptic lineages, differentially fixed for alternative mtDNA *COI* haplogroups and significantly differentiated at microsatellite loci. The mosaic distribution of the two lineages is unusual for a warm‐temperate marine invertebrate with a teleplanic larval stage. The Atlantic lineage was unexpectedly observed as a patch enclosed in the north of the Western Mediterranean Sea between eastern Spain and the French Riviera, and the Mediterranean lineage was found in Macronesian Islands. Although cyto‐nuclear disequilibrium is globally maintained, asymmetric introgression occurs in the Spanish region where the two lineages co‐occur in a hybrid zone. A first interpretation of our results is mito‐nuclear discordance in a stable postglacial hybrid zone. Under this hypothesis, though, the location of genetic discontinuities would be unusual among planktonic dispersers. An alternative interpretation is that the Atlantic lineage, also found in Senegal and Venezuela, has been introduced by human activities in the Mediterranean area and is introgressing Mediterranean genes during its propagation, as theoretically expected. This second hypothesis would add an additional example to the growing list of cryptic marine invasions revealed by molecular studies.

## 1 INTRODUCTION

Patterns of genetic structure in marine systems have been shown to be mainly related to two main factors: dispersal ability and biogeographic barriers (Riginos, Douglas, Jin, Shanahan, & Treml, 2011), to which we should now also append recent human‐related introductions of differentiated lineages by anthropogenic vectors (Rius, Turon, Bernardi, Volckaert, & Viard, 2015). It has long been expected that species with a dispersive larval stage should be less differentiated than species with direct development (Hellberg, 1996; Johnson & Seger, 2001; Palumbi & Baker, 1994), and this has been corroborated by meta‐analyzes of the marine population genetics literature (Kelly & Palumbi, 2009; Selkoe & Toonen, 2011). However, planktonic dispersers have also long revealed pronounced genetic breaks between populations of what was recognized as a single species, and these often collocate with well documented biogeographic boundaries (Gagnaire et al., 2015; Pelc, Warner, & Gaines, 2009; Riginos et al., 2011). Marine biogeographic boundaries often coincide with oceanic fronts thought to impose a physical barrier to dispersal and with strong environmental gradients. As a consequence the genetic differentiation observed at these places has often been explained by reduced connectivity and selection to different environmental conditions (Selkoe, Henzler, & Gaines, 2008). For instance, the Almeria–Oran front that delimits the Mediterranean and Atlantic biogeographic provinces is a hot spot of genetic differentiation in planktonic dispersing species (Patarnello, Volckaert, & Castilho, 2007) and this has been interpreted as a consequence of an efficient barrier to larval dispersal (Galarza et al., 2009). Conversely, the strong salinity gradient observed at the entrance of the Baltic Sea and adaptation to brackish waters have been proposed to explain the genetic differentiation observed in many marine species with high dispersal capabilities between the North Sea and the Baltic Sea (Johannesson & Andre, 2006; Lamichhaney et al., 2012). However, increasing evidence is accumulating suggesting populations each side of these boundaries could more likely be considered cryptic semidifferentiated lineages evolving under reproductive isolation mechanisms. Reproductive isolation might better explain the maintenance of the genetic differentiation, whereas barriers to dispersal and selection against migrants, though contributing to impede connectivity, better explain the position of the genetic breaks rather than their maintenance (Bierne, Welch, Loire, Bonhomme, & David, 2011; El Ayari, Trigui El Menif, Hamer, & Bierne, submitted; Tine et al., 2014). Finally, planktonic dispersers can display pronounced genetic differentiation in nonequilibrium scenarios of recent contact between a native and an introduced lineage by human activities (Heath, Rawson, & Hilbish, 1995). Human‐mediated transport can bring into contact previously isolated, and thus divergent, lineages or species, a phenomenon which can promote admixture at the infraspecific levels and introgression between native and introduced species (Viard, David, & Darling, 2016). When the lineages brought into contact by anthropogenic activities are sibling species, molecular studies thus uncover cryptic species and invasions by a new (cryptic) species. There are a number of cases where seemingly cosmopolitan species were shown to be invasive species and members of complexes of cryptic species, sometimes casting doubts about the native vs. invasive species of these cryptic lineages (Bock, Mac Isaac, & Cristescu, 2012; Bouchemousse, Bishop, Bishop, & Viard, 2016; Dijoux, Viard, & Payri, 2014; Fehlauer‐Ale et al., 2014; Mackie, Darling, & Geller, 2012). We are probably at the dawn to uncover many such cases thanks to the increasing popularization of molecular approaches, and the persistent propagule pressure imposed by unaltered anthropogenic fluxes.

We studied the genetic structure of the red‐mouthed rock shell *Stramonita haemastoma* (Linnaeus, 1767) in the Western Mediterranean Sea and North‐Eastern Atlantic Ocean. *Stramonita haemastoma* is a rocky shore gastropod belonging to the family Muricidae usually found in warm‐temperate waters (Barash & Danin, 1992; Butler, 1985). It displays a typical bentho‐pelagic life cycle. Adults are benthic and have low dispersal capabilities. *Stramonita haemastoma* is gonochoristic. Females lay 20–86 egg capsules each containing 1,700–7,000 eggs (Lahbib, Abidli, & El Menif, 2011). The size of the protoconch (larval shell retained on the adult shell) as well as the duration of larval development in the laboratory allowed to estimate a larval pelagic duration of 2–3 months (Claremont et al., 2011, El Ayari, 2015; Lahbib et al., 2011; Appeltans et al., 2012). Such extremely long‐lived larvae capable of dispersal over long distances are usually called “teleplanic” (Scheltema, 1971). *Stramonita haemastoma* is a complex of species with widespread distribution in the Atlantic Ocean and Eastern Pacific Ocean (Abbott, 1972; Claremont et al., 2011, Clench, 1947). Claremont, Williams, Barraclough, and Reid (2011) conducted a study on the genus *Stramonita* which identified, in addition to the previously recognized outgroup Pacific species *S. delessertiana,* six members in the *Stramonita haemastoma* complex, *S. biserialis* in the South‐Eastern Pacific Ocean, *S. floridana* and *S. canaliculata* in the North‐Western Atlantic Ocean, *S. rustica* and *S. brasiliensis* in the South‐Western Atlantic Ocean, and *S. haemastoma*. The latter is the single representative of the species complex in the Eastern Atlantic, and Mediterranean Sea although molecular data were mostly lacking for Mediterranean samples. The two North American Atlantic species *S. canaliculata* and *S. floridana* were previously described with allozymes and mitochondrial markers (Harding & Harasewych, 2007; Liu, Foltz, & Stickle, 1991) and have been shown to hybridize, suggesting that reproductive isolation cannot be assumed complete between members of the complex.

In this study, we used three newly developed microsatellite loci and mtDNA *COI* sequences to analyze a wide sample of *S. haemastoma* snails in North Africa, Spain, and France. We identified two cryptic lineages with a mosaic distribution and a hybrid zone along Mediterranean Spanish coasts. We argue that such a distribution raises questions about the native *vs*. invasive status of these lineages.

## 2 MATERIALS AND METHODS

### 2.1 Sampling and molecular markers

**Figure 1:**
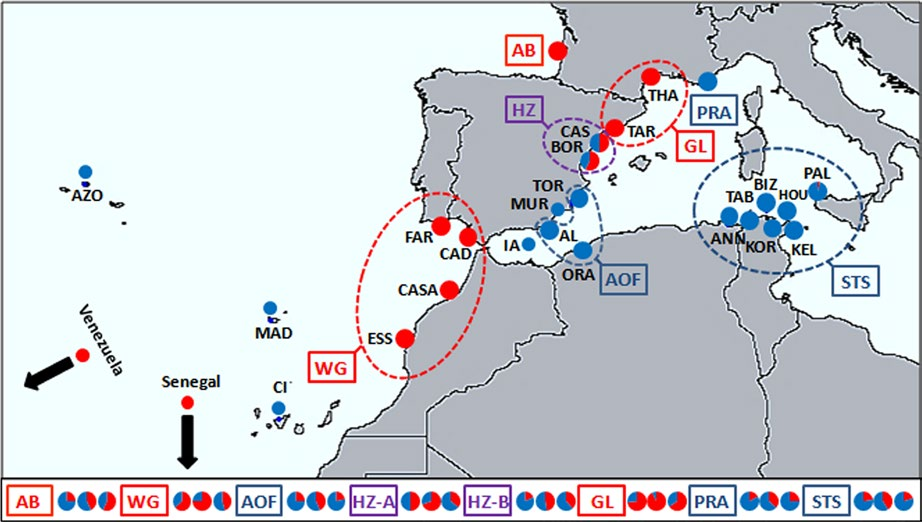
Sampling localities of *Stramonita haemastoma*. Proportion of individuals with haplogroup A (in red) and B (in blue) at the mtDNA *COI* locus for each sampling localities, or sites for which data were available in the literature. Pie charts below the map represent the frequency of the two compound alleles at the three nuclear loci (from left to right: *Strhae3*,* Strhae8,* and *Strhae9*) for groups of samples. HZ‐A and HZ‐B grouped individuals from the two localities of the HZ area according to their haplogroups. Sample names and their GPS positions are given in the Table S1

In addition to mtDNA *COI* sequence data retrieved from GenBank (see Data Analysis), data were obtained from individuals of *Stramonita haemastoma* collected by SCUBA diving in 20 localities; five from the North‐Eastern Atlantic Ocean and 15 from the Mediterranean Sea (Table S1, Figure 1). DNA was extracted from foot tissue using the DNeasy Blood and Tissue kit (Qiagen, Valencia, CA, USA) following the instructions of the manufacturer, with a final elution in 100 μl. DNA concentration was measured for each sample using a NanoDrop8000 Spectrophotometer (Thermo Scientific) and standardized to a DNA concentration of 50 ng/μl. A 586‐bp fragment of the cytochrome oxidase subunit I was amplified with a cocktail (*COI‐2*) of four forward and four reverse primers (Table S2) in the following ratio; 10 pmol/μl, *VF1_t1*:* VF1d_t1*:* LepF1_t1*:* VF1i_t1* (1:1:1:3) or *VR1_t1*:* VR1d_t1*:* LepRI_t1*:* VR1i_t1* (1:1:1:3) and sequenced with *M13* (Ivanova, Zemlak, Hanner, & Hebert, 2007). PCRs with an initial denaturation at 95°C for 5 min followed by 35 cycles (94°C 30 s; 42°C 30 s, 72°C 45 s) followed by a final extension at 72°C for 7 min were performed in 15 μl reaction volume consisting of Dream Buffer 10× (1.5 μl), 5 mM DNTPs (0.6 μl), each primer (0.24 μl), Dream Taq Fermentas (0.06 μl), double‐distilled autoclaved water (10.36 μl), and 2 μl template DNA. Sequence reactions were precipitated using a standard EDTA/ethanol protocol, suspended in 15 μl Hi‐Di formamide 0.2 μl ROX, and sequenced on an ABI 3130XL automated sequencer. The construction of a microsatellite library and the 454‐sequencing was subcontracted to Ecogenics. From the sequences obtained we tested eighteen loci and selected three loci with a good rate of amplification and lack of deviation from Hardy–Weinberg equilibrium. The core microsatellite sequence and primers are provided in Table S3. PCR products were diluted at 1:50 with double‐distilled autoclaved water (2 μl) and were then pooled in a mix of Hi‐Di formamide/ROX 500 size standard (12.8 μl formamide and 0.2 μl ROX); lastly the mixtures were loaded on an ABI 3130XL capillary automated sequencer. GeneMapper^®^ v4.5 software (Applied Biosystems) was used to read the resulting chromatograms.

### 2.2 Data analysis

BIOEDIT V7.1.3.0 was used for sequences alignments. For mtDNA analyzes, we also retrieved sequences from Genbank corresponding to seven geographic samples reported in Claremont et al. (2011) (Table S1). Aligned sequences were then used to construct a Neighbor‐Joining tree with MEGA software v6. Phylogenetic supports were tested with bootstrap tests (500 bootstraps). For the implementation of a minimum spanning network, we used PopArt 1.7 (Bandelt, Forster, & Röhl, 1999). The DnaSP V5.10.1 software (Librado & Rozas, 2009) was used to calculate the number of haplotypes (h), haplotype diversity (hd), nucleotide diversity (π), average number of nucleotide substitutions per site between clusters (Dxy) and net number of nucleotide substitutions per site between clusters (Da). For microsatellites, allele frequencies, genotype frequencies, average number of alleles/locus, and pairwise *Fst* were obtained using Genetix software v4.0.5.2 (Belkhir, Borsa, Chikhi, Raufaste, & Bonhomme, 2002). Genetix was also used to perform a correspondence analysis (CA) on individual genotypes. Linkage disequilibrium was tested with an exact test in genepop'007 (Rousset, 2008). The genetic structure was examined using STRUCTURE 2.3.4 with 10.000 burn‐in iterations and 100.000 iterations (Falush, Stephens, & Pritchard, 2003). We used the admixture model. We let *K* range from 1 to 9 with 100 replicated runs for each *K*. Results gathered from those runs were tested with STRUCTURE HARVESTER (Earl, 2012). The number of clusters was chosen based on Evanno's Δ*k* value (Evanno, Regnaut, & Goudet, 2005). Data generated by STRUCTURE were also analyzed using CLUMPAK, which compares all runs at each value of *K* in order to identify optimal clustering scenarios (Kopelman, Mayzel, Jakobsson, Rosenberg, & Mayrose, 2015).

## 3 RESULTS

### 3.1 Genetic variability

**Figure 2:**
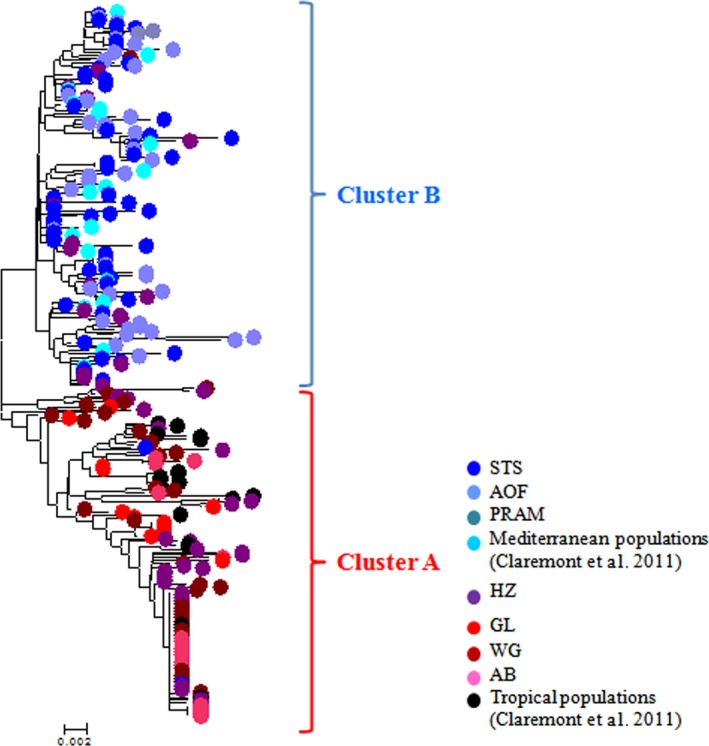
Neighbor‐joining tree of mtDNA *COI* sequences from *Stramonita haemastoma* samples. STS (Annaba, Tabarca, Bizerte, Korbous, Kelibia, Houaria, and Palermo), AOF (Oran, Almeria, Torreveija), PRAM (Pramousquier), Mediterranean populations (Murcia, Alborán Island, Azores, Canary Islands, Madeira), HZ (Castellon and Borriana), GL (Thau and Tarragona), WG (Casablanca, Essaouira, Cadiz, Faro), AB (Arcachon Bay), Tropical populations (Venezuela and Senegal). See Figure 1 for site locations

**Figure 3:**
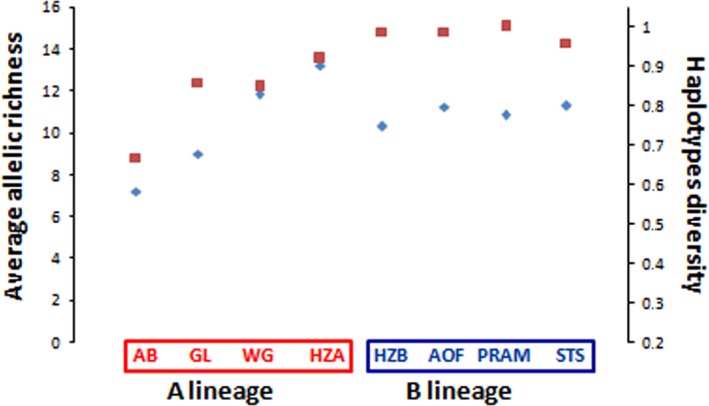
Average allelic richness at microsatellites loci (blue diamonds) and haplotype diversity in mtDNA *COI* sequences (red square) in A and B lineage populations

The analysis of the mitochondrial diversity has revealed 167 haplotypes and a high haplotypic and nucleotide diversity (hd = 0.976 and π = 0.016), over the whole dataset. Two mitochondrial clusters of sequences, hereafter named haplogroups, were clearly distinguished in the Neighbor‐Joining tree (Figure 2) and in the minimum spanning network (Fig. S1). Haplogroups A and B were defined according to their phylogenetic relationships, and their frequency computed for each study locality (Figure 1). Average and net nucleotide divergence measures between the five Atlantic populations fixed for the A haplogroup and the 10 Mediterranean populations fixed for the B haplogroup were high (Dxy = 0.022, Da = 0.012) as compared to the divergence within haplogroups (A: Dxy = 0.009, Da = 0; B:Dxy = 0.010, Da = 0). A total of 62 alleles were identified using three microsatellites (22 size‐alleles at the locus *Strhae3*, 19 at *Strhae8* and 21 at *Strhae9*). The null hypothesis of linkage equilibrium was never rejected with exact tests. Allele frequencies are presented in Table S4. Because of unevenness of sample sizes, samples were pooled into groups according to their geographic proximity as described with the ellipses in Figure 1, after having verified the genetic homogeneity with an individual‐based clustering method implemented in STRUCTURE (see results below). The average allelic richness at microsatellite loci for each locality or group is represented together with the mtDNA *COI* haplotype diversity in Figure 3. Comparison among loci can be hampered by variation in diversity (Hedrick, 1999; McDonald, 1994), especially when the differentiation is strong, as in hybrid zones, and the diversity is high, as for microsatellites. To circumvent this problem, one can use alternative statistics such as Jost's D (Jost, 2008) or related measures (Meirmans & Hedrick, 2011), but in the case of a contact between two lineages pooling alleles is the most straightforward procedure (Barton, 2000; McDonald, 1994). Microsatellite alleles were combined to construct bi‐allelic loci: alleles were pooled into two compound A and B alleles, according to their frequencies in Atlantic populations fixed for the A haplogroup and Mediterranean populations fixed for the B haplogroup (see Table S4). The same grouping of alleles was obtained when using the allele coordinates in a correspondence analysis as explained in Bierne et al. (2003). The frequency of the compound A allele and compound B allele is reported in Figure 1.

### 3.2 Distribution of the mitochondrial lineage across *Stramonita haemastoma* populations

The group of samples from the area around the Siculo‐Tunisian Strait (STS), including samples from Tabarca, Bizerte, Korbous, Kelibia and Houaria (Tunisia), Annaba (Algeria), and Palermo (Italy) were nearly exclusively composed of cluster B haplotypes at the mitochondrial locus, with the exception of two individuals found in Palermo. Samples from the area around the Almeria–Oran Front (AOF) including Torreveija and Almeria (Spain), and Oran (Algeria) were fixed for the B haplogroup. Finally, our sample from the French Riviera in Pramousquier (France) was also composed of cluster B haplotypes. In addition, data from Claremont et al. (2011) showed that the B haplogroup is also found in Murcia, Alborán Island, and Macaronesian islands (Figure 1). The group of samples from the area west of Gibraltar (WG), including Casablanca and Essaouira (Morocco), Cadiz (Spain), and Faro (Portugal) were fixed for the A haplogroup. Similarly, the sample from the Atlantic coast of France in Arcachon Bay was also entirely composed of cluster A haplotypes. Surprisingly, cluster A haplotypes were also found at high frequency in the North‐Western Mediterranean samples and fixed in Thau (France) and Tarragona (Spain). Again data from Claremont et al. (2011) were used, and the A haplogroup was also found in tropical waters in Senegal and in Venezuela (Figure 1). Finally, and importantly, two samples from the Valencia area along Eastern Spanish coasts (Castellon and Borriana) were found to be a balanced mixture of both mitochondrial clusters.

### 3.3 Cyto‐nuclear disequilibrium in *Stramonita haemastoma* populations

**Figure 4:**
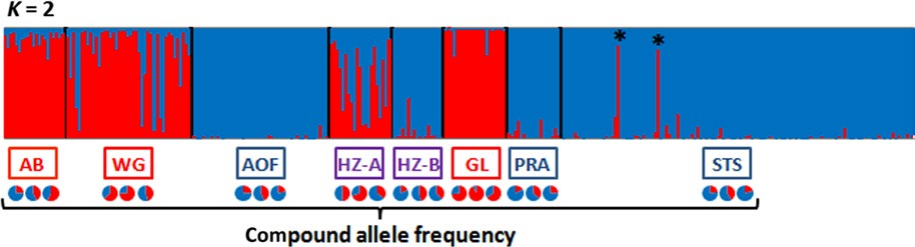
Bar plot of the estimated cluster membership fraction (Q) estimated by STRUCTURE for *K* = 2, which received highest support from Evanno's Δ*k* (*) individuals carrying a cluster A mtDNA haplotype in the Palermo sample. Pie charts below the map represent the frequency of the two compound alleles at the three nuclear loci (*Strhae3*,* Strhae8,* and *Strhae9*) for each studied group of populations

**Figure 5:**
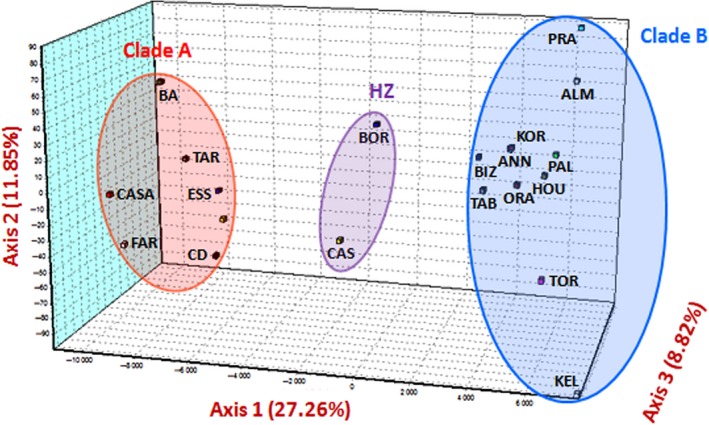
Correspondence analysis (CA) at mtDNA *COI* and nuclear loci (*Strhae3*,* Strhae8,* and *Strhae9*). Cluster A, B, and samples of the Spanish hybrid zone are circled separately. Sample names are given in Table S1

The analysis of the three nuclear microsatellite loci with STRUCTURE revealed the most parsimonious number of clusters was *K* = 2, as supported by Evanno's Δ*k* (Fig. S2) and the exploration of outputs with CLUMPAK that revealed two well defined clusters and no minor cluster with *K* = 2. The STRUCTURE output is presented in Figure 4. The result obtained is mostly concordant with the mtDNA data, with populations fixed for the A haplogroup assigned to a given genetic cluster (red cluster in Figure 4), that we accordingly called cluster A, and populations fixed for the B haplogroup assigned to the other genetic cluster (blue cluster in Figure 4), accordingly called cluster B. The differentiation between both clusters is also visible in the correspondence analysis (CA), which shows that axis 1 differentiates cluster A from cluster B (Figure 5). However, the genetic differentiation was much lower at nuclear loci than at the mtDNA locus (Table S5). The average nuclear *Fst* between samples fixed for the mtDNA haplogroup A and samples fixed for the mtDNA haplogroup B was 0.02 (permutation tests *p* < .001), while no significant nuclear genetic differentiation was found within these entities with the single exception of the Arcachon Bay sample (belonging to lineage A) that proved to be slightly differentiated to other lineage A populations (*Fst* = 0.04, *p* < .001). This genetic difference of Arcachon Bay sample with other samples from cluster A was also revealed by the STRUCTURE analyzes for *K* = 3 (Fig. S2). With the exception of Arcachon Bay within cluster A, genetic panmixia was the rule within cluster whatever the distance between sampling sites.

The structure observed at nuclear loci can also be viewed with the frequency of the compound lineage‐specific alleles reported in Figures 1 and 4 for each study localities. By construction the frequency of the compound A‐allele is higher in samples fixed for the A haplogroup, and the frequency of the compound B‐allele higher in samples fixed for the B haplogroup. The two samples in Castellon and Borriana (CAS and BOR) showing mitochondrial admixture displayed intermediate nuclear allele frequencies and nuclear admixture in the STRUCTURE analysis. This pattern may be indicative of hybridization or introgression. In order to better examine the mito‐nuclear disequilibrium maintained in these putative hybrid populations, individuals from these two localities were sorted into two groups named HZ‐A and HZ‐B according to their mitochondrial haplotype (A and B, respectively) before being processed in the STRUCTURE analysis (Figure 4) and to display the microsatellite compound allele frequencies in Figure 1 and below the STRUCTURE plot in Figure 4. Individuals with a B mtDNA haplotype have a homogeneously high proportion of cluster B ancestry (Figure 4) as were individuals sampled in allopatric populations (i.e., populations where only one haplogroup was found). Individuals with an mtDNA A haplotype tend to have a higher proportion of cluster A ancestry but some have a higher proportion of cluster B ancestry than others (Figure 4). Interestingly the two individuals with A haplotype sampled in Palermo in the STS area also have a high proportion of cluster A ancestry (stars in Figure 4), showing that the mito‐nuclear disequilibrium extends to this population. Finally, a few (~10%) individuals of the Western Gibraltar strait area also have a higher proportion of cluster B ancestry than the others while all carrying A haplotypes.

**Figure 6:**
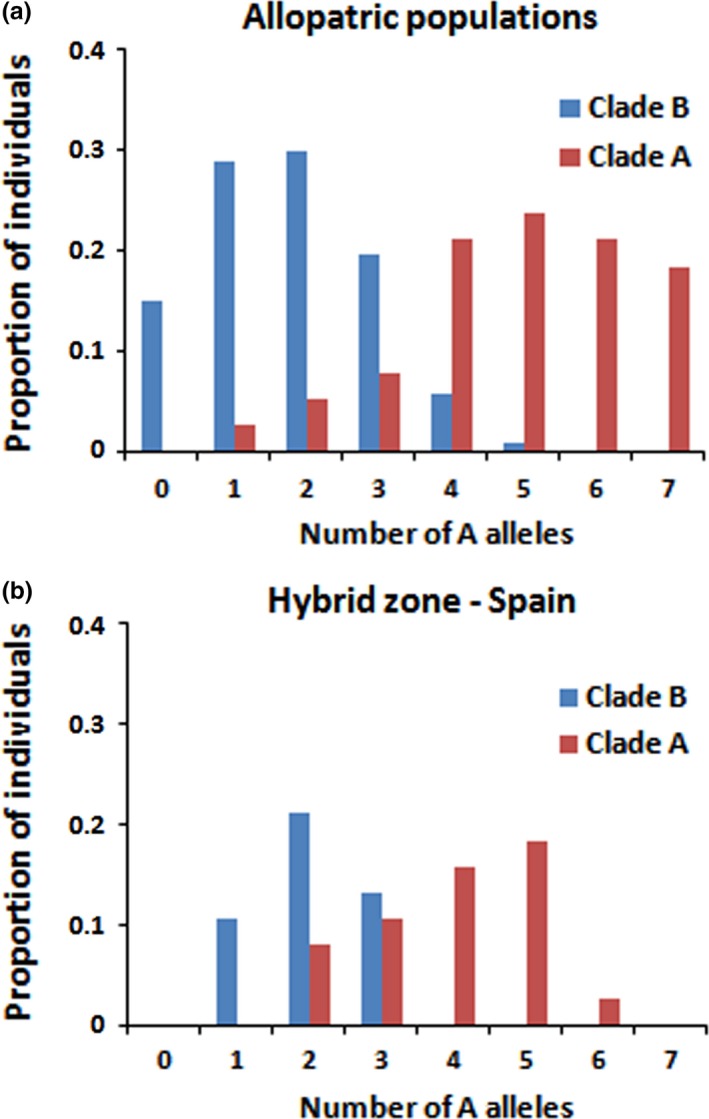
Distribution of the hybrid index, the number of lineage A alleles per individual, in (a) allopatric populations and (b) populations of the Spanish hybrid zone

In order to illustrate the genetic compositions of samples with or without mitochondrial lineages mixture, we have plotted in Figure 6 the distribution of the hybrid index, which is for each individual the number of A alleles summed over the mtDNA locus and the three nuclear microsatellite loci. Figure 6a shows that the two distributions overlap little when considering allopatric populations. The overlapping is due to shared ancestral polymorphisms and/or introgression at nuclear loci, and possibly to a few hybrids (e.g., in the WG area where some A mtDNA haplotype carrying individuals have a high proportion of B ancestry at microsatellite markers). In the populations CAS and BOR of the Spanish putative hybrid zone (HZ in figures), the overlapping is stronger (see Figure 6b), with the HI distribution of A‐cluster individuals shifted to lower HI values and to a lesser extent the HI distribution of B‐cluster individuals shifted to higher HI values. This result highlights hybridization and local asymmetric introgression in the Spanish hybrid zone, which was already visible in the STRUCTURE plot in Figure 4.

## 4 DISCUSSION

Our mito‐nuclear genetic analysis of the teleplanic dispersal snail *S. haemastoma* in the Western Mediterranean Sea and Eastern Atlantic Ocean has revealed (i) two cryptic mitochondrial lineages also differentiated at three nuclear microsatellite markers, (ii) an unexpected mosaic distribution of the two lineages with a surprising patch of the Atlantic lineage in the North‐Western part of the Occidental basin in the Mediterranean Sea and (iii) hybridization between the two lineages when found in sympatry. There is little need to discuss at length the discovery of cryptic lineages with molecular markers as this observation is pervasive in the sea (Appeltans et al., 2012; Knowlton, 1993; Pante, Abdelkrim, et al., 2015; Pante, Puillandre, et al., 2015). However, the geographic distribution of molecularly identified cryptic lineages is usually compatible with vicariant divergence between well recognized biogeographic regions, especially when the divergence is recent and hybridization happens in the contact zone. Here, the two discovered semi‐isolated lineages have an unusual distribution and meet in a hybrid zone the position of which is novel for a planktonic dispersing marine species. We will here discuss the two possible scenarios that can explain the mosaic distribution and genetic composition of hybrid zone samples: (i) a stable postglacial distribution with mito‐nuclear discordance or (ii) a recent invasion of the A lineage within the distribution range of the B lineage.

### 4.1 Hypothesis 1: A stable postglacial hybrid zone with mito‐nuclear discordance, and a patch of cold water adapted Atlantic‐derived populations blocked in the North‐Western Occidental Mediterranean Sea

The geography of the Mediterranean Sea, perpendicular to north‐south population displacements due to glacial oscillations, has been prone to trap cold‐adapted species in pockets of cold waters in the north, in the Gulf of Lion in the Occidental basin, in the northern Adriatic Sea and the Black Sea. The flounder *Plathichthys flesus* (Borsa et al., 1997), the sprat *Sprattus sprattus* (Debes, Zachos, & Hanel, 2008), and the planktonic chaetognatha *Sagitta setosa* (Peijnenburg, Breeuwer, Pierrot‐Bults, & Menken, 2004) are examples of species with a discontinuous geographic distribution in the Northern Mediterranean Sea. For those three examples, genetic divergence between Atlantic and Mediterranean populations and between Northern Mediterranean pockets has been revealed. These observations are compatible with isolation after postglacial warming. The same explanation could be given to the results obtained with *S. haemastoma*. The absence of genetic differentiation between Atlantic and North‐Western Mediterranean populations of the A lineage is indeed plausible with only a few thousand years of isolation (Faure, David, Bonhomme, & Bierne, 2008). We can also imagine that the 3 months long teleplanic larvae phase could have permitted the implantation of the A lineage in the Mediterranean Sea by long‐distance colonization of an empty patch. If the Spanish hybrid zone is a stable postglacial contact zone, then the observation that A mtDNA haplogroup carrying individuals tend to sometimes have a high proportion of B lineage ancestry at the nuclear loci would be well explained by the widespread observation of mito‐nuclear discordance in hybrid zones (Toews & Brelsford, 2012). A finer sampling grain along the coast would be required to better describe this phenomenon. The two sexes dispersing similarly in a planktonic disperser, and assuming under the postglacial hybrid zone hypothesis that the zone did not move recently, the best explanation would be the existence of cyto‐nuclear incompatibilities (Burton, Pereira, & Barreto, 2013; Chou & Leu, 2015).

However, a series of evidence makes this hypothesis unlikely. First, we are not aware of a species/lineage with a discontinuous distribution in the north Atlantic and cold water pockets of the Northern Mediterranean Sea that would have remained in contact with a sister nonreproductively isolated (and thus hybridizing) lineage distributed elsewhere in the Mediterranean Sea. Furthermore, in each of these other examples of discontinuous distribution, the geographical range of the distribution in the Atlantic Ocean is preponderantly north to the Cantabrian Sea, in accordance with a temperate water preferendum. On the contrary, the distribution of the A lineage of *S. haemastoma* corresponds to warm‐temperate preferendum. It is south to the Cantabrian Sea (Claremont et al., 2011), with the A lineage found in abundance in Portugal and Morocco, and according to the data of Claremont et al. (2011) found in tropical waters in Senegal and Venezuela.

A last surprising observation is the location of the hybrid zone itself. Postglacial secondary contact zones between Atlantic and Mediterranean lineages have always been detected at the Almeria–Oran front in planktonic dispersing marine species (Patarnello et al., 2007). One explanation is that the barrier to larval dispersal and/or the environmental boundary at the Almeria–Oran front have acted as a trap for hybrid zones (Bierne et al., 2011). The Spanish hybrid zone of *S. haemastoma* is therefore at an unusual place. In addition, the genetic break between the Gulf of Lion and the French Riviera would also be an unusual result in a planktonic disperser. Genetic differentiation has often been reported within the Mediterranean occidental basin in restricted dispersers (Boissin, Hoareau, Féral, & Chenuil, 2008; Rastorgueff, Chevaldonné, Arslan, Verna, & Lejeusne, 2014), but rarely in species with larval dispersal. Mediterranean corals showed strong population differentiation in this region (Ledoux et al., 2010; Masmoudi et al., 2016; Mokhtar‐Jamai et al., 2011), but the effective dispersal distance of these species is debated, and cryptic semi‐isolated ecotypes are suspected (Aurelle et al., 2017). Only the edible sea urchin *Paracentrotus lividus* shares a long larval duration and within‐basin genetic differentiation that was also found unexpected by (Penant, Aurelle, Feral, & Chenuil, 2013), although the genetic structure was not as strong and clear‐cut as found here in *S. haemastoma*. Besides we would emphasise that while uncovering genetic breaks at unusual geographic locations, our samples from Almeria, together with the data of Claremont et al. (2011) from the Island of Alboran, also suggest that the Almeria–Oran front has no effect on the genetic structure of the B lineage.

### 4.2 Hypothesis 2: Recent invasion of the A lineage within the range of the B lineage

The mosaic distribution of the two lineages and the localization of the hybrid zone in Spain suggest that human‐mediated transports could be proposed as an alternative explanation to natural distribution. The genetic results could indeed be explained by a biological introduction of the A lineage into the natural distribution range of the B lineage. It is now well documented that human activities substantially alter biogeographical patterns (e.g., (Capinha, Essl, Seebens, Moser, & Pereira, 2015), and the marine realm is no exception as pointed out by numerous cases of cryptogenic species—that is species of unknown native *vs*. introduced status sensu (Carlton, 1996; Haydar, 2012) and cosmopolitan invaders crossing biogeographic barriers (e.g., the bryozoans *Watersipora* spp. (Mackie et al., 2012), the red algae *Asparagopsis* spp. (Dijoux et al., 2014), or the tunicate *Ciona robusta* (Bouchemousse, Bishop, et al., 2016)). Marine introductions have been occurring since the end of the 19th century at an increasing rate, in particular in the Mediterranean Sea (Galil et al., 2014; Katsanevakis, Zenetos, Belchior, & Cardoso, 2013). The Thau lagoon in the Gulf of Lion and the Arcachon Bay is recognized hot spots of marine invasion (Mineur, Belsher, Johnson, Maggs, & Verlaque, 2007; Verlaque, Auby, & Belsher, 2008). Species were introduced for aquaculture purpose like the Indo‐pacific clam *Ruditapes philippinarium* (Chiesa et al., 2014; Gosling, 2003) or the Japanese oyster *Magallana gigas* (formerly known as *Crassostrea gigas*) (Grizel & Héral, 1991; Ruesink et al., 2005). Species were also introduced, for most of them, accidentally with shellfish stocks, like *Grandidierella japonica* introduced in the Arcachon Bay with oysters (Lavesque et al., 2014; Mineur et al., 2007) or *Sargassum miticum* (Engelen et al., 2015). *Stramonita haemastoma* is an oyster predator which is usually found on oyster beds (Rothschild et al., 1994). Aquaculture is the most likely vector of introduction for this species. Identified non‐native species might indeed be the tip of the iceberg. It is most likely that a vast number of unnoticed (cryptic) invasions occurred in the past, promoting secondary contacts between previously isolated lineages or species (Viard et al., 2016). In addition, an increasing number of studies in marine invasion genetics emphasize invasions by cryptic lineages previously undistinguishable from the native lineage (Geller, Darling, & Carlton, 2010). Such a situation has been described for instance within the species complex *Ciona intestinalis* (Zhan, Macisaac, & Cristescu, 2010), and in particular between the two species now accepted, *C. intestinalis* and *C. robusta,* in the Western English Channel (see (Bouchemousse, Haag‐Liautard, Bierne, & Viard, 2016), and references therein). In order to identify an introduction, one ideally needs to identify the source of the invasion, and, in addition, the geographic gap between the source and the invasion area needs to be obviously too far for natural connectivity or separated by some barriers. However, in the case of *S. haemastoma,* the source is very difficult to identify, and the existence of a long‐lived larva may promote long‐distance dispersal. If introduced, putative sources could be Atlantic coasts (NE Atlantic or Western Africa), South America, or South‐West Africa. Small scale invasion from the Atlantic coasts has never been reported in another bentho‐pelagic marine species but as explained above is difficult to demonstrate as it is difficult to refute a natural postglacial redistribution of vicariant lineages. Claremont et al. (2011) found the A lineage in Venezuela and suggested a transoceanic colonization, the widespread distribution in Eastern Atlantic and apparently limited distribution in America, and the reduced genetic diversity in Venezuela indicating an East to West migration by the South Equatorial Current (Boehm et al., 2013; Claremont et al., 2011; Lapègue et al., 2002). An invasion from America is therefore considered unlikely. Finally, South‐West Africa represents a promising area of investigation as *S. haemastoma* has been reported in this area (Clench, 1947; Penrith & Kensley, 1970). However, it is far from clear if the equatorial region represents a barrier to dispersal in this teleplanic species that seems to tolerate tropical waters quite well.

Rather than identifying the route of invasion, one can also gain some evidence in accordance with a cryptic invasion in the genetic diversity and genetic structure. As compared to a species within its native range, an introduced marine species is expected to display a low frequency or absence of private (endemic) haplotypes, weaker genetic structure between introduced populations, and more importantly a lack of concordance between the phylogeny and the geographical distribution of the haplotypes (i.e., phylogeographic discordance) (Viard et al., 2016 and references herein). For example, conversely to what have been observed in many native Chilean species, no genetic differentiation was found between populations of *Ciona robusta* located on both sides of the recognized biogeographical boundary at 30–33°S. This pattern supported the non‐native status of this species in Chile (Bouchemousse, Bishop, et al., 2016). Similarly, the gastropod *Tritia neritea* displays a strong phylogeographic structure over its native range, in the Mediterranean Sea, but shows almost no structure and high level of admixture in its introduction range along the French Atlantic coasts (Simon‐Bouhet, Garcia‐Meunier, & Viard, 2006). Here, such discordance is observed with the haplogroup A spread over two very distinct biogeographic provinces (the Lusitanian and Mediterranean provinces, sensu (Spalding et al., 2007). We also observed an unexpected location of the hybrid zone as compared to what has been documented in other Mediterranean marine native species. These two arguments support a non‐native status of the lineage A in this area. In addition, we observed an asymmetric pattern of introgression which resembles well the theoretical prediction of native alleles introgression into the invading background (Currat, Ruedi, Petit, & Excoffier, 2008). Finally, the presence of two lineage A individuals, with strong mito‐nuclear and intranuclear disequilibrium (Figure 4) in Palermo, an area dominated by the B lineage, suggests a very recent arrival of the A lineage in Sicily, or hybridization rates would be lower in Sicily than in Eastern Spain. We acknowledge that none of the above arguments allowed to clearly dismiss hypothesis 1 and thus to conclude whether the mosaic distribution is due to human‐mediated introductions. New genetic data can help. Increasing the number of loci analyzed by taking advantage of new high‐throughput sequencing method will help to better understand the genetic differentiation in this system (Viard et al., 2016). In addition, broadening the geographic sampling of this species (in contact zones, in South‐West Africa and Central America) is also an important issue.

## 5 CONCLUSION

We have discovered two cryptic lineages in North‐Eastern Atlantic and Mediterranean populations of the teleplanically dispersing marine snail *S. haemastoma*, with an unusual mosaic distribution. This mosaic distribution could result from human‐mediated introduction: The unexpected location of the sympatric area, the patchy, and restricted distribution of the lineage A and the asymmetric introgression pattern support the hypothesis that the A lineage is invading in the Mediterranean Sea. We were, however, not able to fully dismiss the alternative hypothesis that the mosaic distribution results from postglacial population displacement, the asymmetry of introgression in the hybrid zone being then a consequence of mito‐nuclear discordance in a stable hybrid zone. Our results clearly call for deeper investigation: A broader geographic and genomic sampling together with a monitoring of the distribution could allow disentangling of the two hypotheses. In any case, *S. haemastoma* proves an interesting model species to better understand the connectivity of planktonic dispersers in the Occidental basin of the Mediterranean Sea.

## CONFLICT OF INTEREST

None declared.

## AUTHOR CONTRIBUTIONS

N.B and T.E.A designed the study. T.E.A, N.T.E.M, C.S, and D.C sampled *S. haemastoma*. T.E.A conducted the molecular laboratory work. T.E.A and N.B analyzed the data. T.E.A and N.B. wrote the manuscript. F.V revised and commented the article.
