# Supplementary material for: Unexpected mosaic distribution of two hybridizing sibling lineages in the teleplanically dispersing snail Stramonita haemastoma suggests unusual postglacial redistribution or cryptic invasion
Source: Ecol Evol. 2017 Sep 25;7(21):9016–26. doi: 10.1002/ece3.3418 (PMC5689492; doi:10.1002/ece3.3418)
Supplement: Supplementary file 1 [file ECE3-7-9016-s001.docx]

**Supplementary Data: Unexpected mosaic distribution of two hybridizing sibling lineages in the teleplanically dispersing snail *Stramonita haemastoma sensu stricto* suggests cryptic invasion**

**Fig S1.** Minimum spanning network based in mtDNA *COI* sequences from *Stramonita haemastoma* samples.

**Fig S2.** Plot showing Evanno's Δk with one peak corresponding to K = 2.


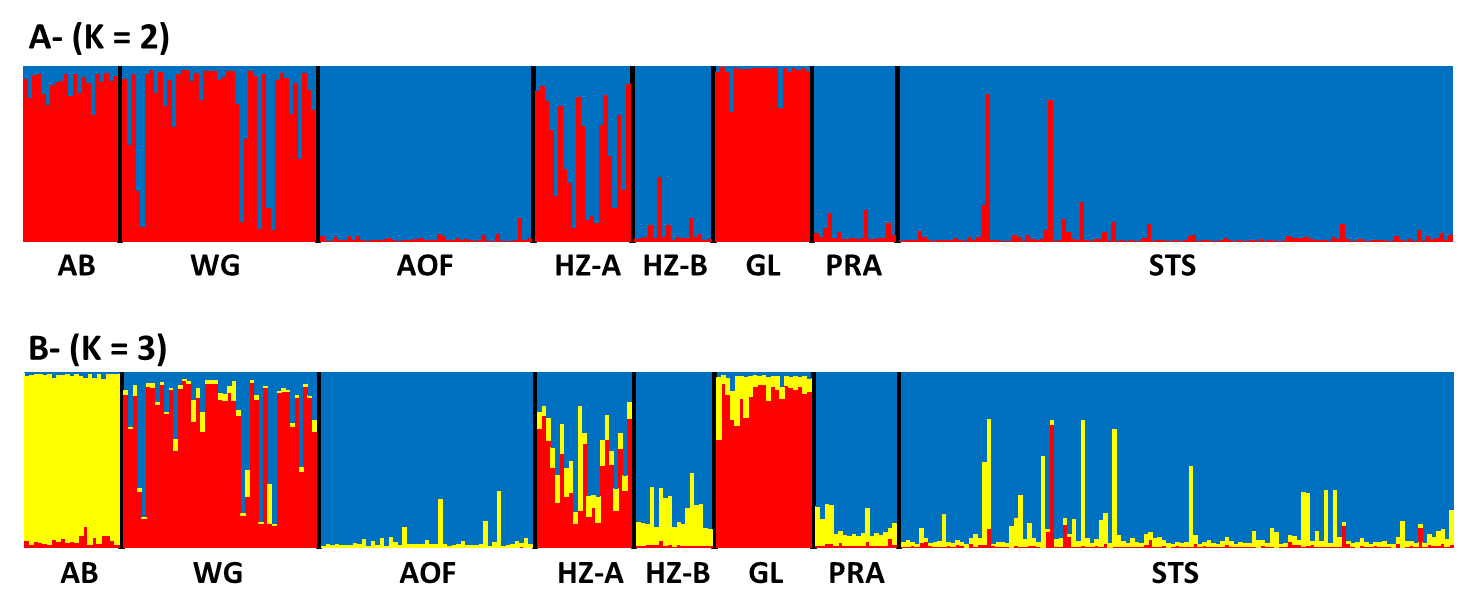


**Fig S2.** Results of the STRUCTURE analysis shown for both K=2 and K=3. Results are shown per group of samples as defined in Figure 1 in the main text.

|  |  |  |  | GPS coordinates | |
| --- | --- | --- | --- | --- | --- |
| Abbreviation | **Sample name** | **Sample size** | **Reference** | **Latitude** | **Longitude** |
| AB | Arcachon Bay | 34 | Present study | 44°35'33.36" N | 1°12'52.56"W |
| FAR | Faro | 6 | Present study | 36°59'19.38" N | 7°59'1.30"W |
| CAD | Cádiz | 30 | Present study | 36°20'58.61" N | 6°15'4.91"W |
| CASA | Casablanca | 15 | Present study | 33°48'37.83" N | 7°11'7.87" W |
| ESS | Essaouira | 28 | Present study | 31°41'32.87" N | 9°48'56.38" W |
| PRA | Pramousquier | 30 | Present study | 43°9'10.507" N | 6°26'54.38" E |
| THA | Thau | 26 | Present study | 43°23'36.03" N | 3°42'10.74" E |
| TAR | Tarragona | 2 | Present study | 41°2'51.12" N | 1°15.15.49" E |
| CAS | Castellon | 15 | Present study | 39°54'10.88" N | 0°1'.13.07" E |
| BOR | Borriana | 29 | Present study | 39°49'5.85" N | 0°4'39.31" W |
| TOR | Torreveija | 20 | Present study | 37°56'22.81" N | 0°41'.929" W |
| ALM | Almeria | 5 | Present study | 36°4'44.67" N | 2°27'23.06" E |
| ORA | Oran | 32 | Present study | 35°54'5.07" N | 0°19'54.95"W |
| ANN | Annaba | 22 | Present study | 36°53'53.66" N | 7°46'17.15" E |
| TAB | Tabarca | 12 | Present study | 37°30'51.27" N | 9°52'9.94" E |
| BIZ | Bizerta | 47 | Present study | 36°50'9.70" N | 11°6'4.81" E |
| KOR | Korbous | 30 | Present study | 36°4'0.65" N | 10°34'1.02" E |
| KEL | Kélibia | 15 | Present study | 38°7'34.60" N | 12°47'37.40"E |
| HOU | Houaria | 26 | Present study | [37.1'59.90" N](javascript:void(0)) | 11°3'33.73"E |
| PAL | Palermo | 30 | Present study | 43°19'57.87" N | 3°36'44.69" E |
| SEN | Senegal | 12 | Claremont et al. (2011) | 14°17'22.63" N | 16°571.99" W |
| VEN | Venezuela | 7 | Claremont et al. (2011) | 10°32'53.74" N | 66°17'41.91" W |
| IA | Isla de Alborán | 5 | Claremont et al. (2011) | 35°52'45.45" N | 3°5'57.62" W |
| MUR | Murcia | 3 | Claremont et al. (2011) | 37°50'51.09" N | 0°44'40.22" W |
| AZO | Azores | 7 | Claremont et al. (2011) | 38°39'7.82" N | 27°13'41.64" W |
| MAD | Madeira | 2 | Claremont et al. (2011) | 32°40'15.52" N | 17°4'2.48" W |
| CI | Canary Islands | 6 | Claremont et al. (2011) | 28°23'26.11" N | 16°41'.43.82"W |

**Table S1.** *Stramonita haemastoma* sample location.

**Table S2.** Sequences (5’–3′) of primers used for the *Cytochrome Oxydase subunit I*.

| **COI-2** | **Primersequence (5’–3′)** | **References** |
| --- | --- | --- |
| LepF1_t1 | *TGTAAAACGACGGCCAGTATTCAACCAATCATAAAGATATTGG* | Hebert et al.(2004) |
| VF1_t1 | *TGTAAAACGACGGCCAGTTCTCAACCAACCACAAAGACATTGG* | Ivanova et al.(2006) |
| VF1d_t1 | *TGTAAAACGACGGCCAGTTCTCAACCAACCACAARGAYATYGG* | Ivanova et al. (2006) |
| VF1i_t1 | *TGTAAAACGACGGCCAGTTCTCAACCAACCAIAAIGAIATIGG* | Ivanova et al.(2006) |
| LepRI_t1 | *CAGGAAACAGCTATGACTAAACTTCTGGATGTCCAAAAAATCA* | Hebert et al. (2004) |
| VR1d_t1 | *CAGGAAACAGCTATGACTAGACTTCTGGGTGGCCRAARAAYCA* | Ivanova et al.(2006) |
| VR1_t1 | *CAGGAAACAGCTATGACTAGACTTCTGGGTGGCCAAAGAATCA* | Ward et al.(2005) |
| VR1i_t1 | *CAGGAAACAGCTATGACTAGACTTCTGGGTGICCIAAIAAICA* | Ivanova et al.(2006) |

**Table S3.** Sequences of primers used for microsatellites loci.

| **Locus** | **Repeat motif** | **Dye color** | **Forward Primer Sequence** | **Reverse Primer Sequence** | **Annealing temperature (°C)** |
| --- | --- | --- | --- | --- | --- |
| *Strhae3* | (*CCTA*) | FAM | *ACCCACCTCTACCTAACTGC* | TGGACTGGAGATCGAATGGG | 60°C |
| *Strhae8* | (*CTGT*) | NED | *CTTCCTACACCACCGGAGAG* | AGCAGATTGCAGATAGACATGC | 58°C |
| *Strhae9* | (*ACTT*) | HEX | *TTCTGGCACTCCCCCATTTC* | TCTGCTTCCAACATACTGTGC | 54°C |

**Table S4.** Allelic frequencies at *COI-2*, *Strhae3*, *Strhae8* and *Strhae9*. Compound A alleles: A, (A_1_-A_11_), (A'_1_- A'_13_), (A"_1_- A"_8_); Compound B Alleles: M, (M_0_- M_11_), (M'_0_- M'_6_), (M"_0-_ M"_12_).

| **Locus** | **Allele** | **AB** | **Faro** | | **CAD** | | **CASA** | | **ESS** | | **THA** | | **TAR** | | **CAS** | | **BOR** | | **TOR** | | **ALM** | | **ORA** | | **ANN** | | **TAB** | | **BIZ** | | **KOR** | | **KEL** | | **HOU** | | **PAL** | | **PRA** | |  |
| --- | --- | --- | --- | --- | --- | --- | --- | --- | --- | --- | --- | --- | --- | --- | --- | --- | --- | --- | --- | --- | --- | --- | --- | --- | --- | --- | --- | --- | --- | --- | --- | --- | --- | --- | --- | --- | --- | --- | --- | --- | --- |
| ***COI-2*** |  |  |  | |  | |  | |  | |  | |  | |  | |  | |  | |  | |  | |  | |  | |  | |  | |  | |  | |  | |  | |  |
| ***100*** | A | 1 | 1 | | 1 | | 1 | | 1 | | 1 | | 1 | | 0.6 | | 0.6333 | | − | | − | | − | | − | | − | | − | | − | | − | | − | | 0.0769 | | − | |  |
| ***200*** | M | − | − | | − | | − | | − | | − | | − | | 0.4 | | 0.3667 | | 1 | | 1 | | 1 | | 1 | | 1 | | 1 | | 1 | | 1 | | 1 | | 0.9231 | | 1 | |  |
| ***Strhae3*** |  |  |  | |  | |  | |  | |  | |  | |  | |  | |  | |  | |  | |  | |  | |  | |  | |  | |  | |  | |  | |  |
| ***80*** | M_1_ | − | − | | − | | − | | − | | − | | − | | 0.0333 | | 0.0167 | | 0.0385 | | − | | − | | − | | 0.05 | | 0.0208 | | − | | − | | − | | 0.0167 | | − | |  |
| ***84*** | M_2_ | − | 0.0833 | | − | | − | | − | | 0.0192 | | − | | 0.1 | | − | | 0.1923 | | − | | − | | − | | 0.25 | | 0.0417 | | − | | 0.4333 | | − | | − | | − | |  |
| ***86*** | M_3_ | 0.0147 | − | | 0.0333 | | 0.0333 | | 0.0625 | | − | | 0.25 | | − | | 0.25 | | 0.0385 | | 0.5 | | 0.1333 | | 0.0357 | | − | | 0.0208 | | 0.3182 | | − | | 0.22 | | 0.1333 | | 0.3333 | |  |
| ***90*** | M_4_ | 0.4412 | − | | − | | 0.0333 | | 0.125 | | 0.0192 | | − | | 0.0333 | | 0.1 | | 0.0385 | | 0.2 | | 0.1 | | 0.5 | | − | | 0.1458 | | − | | 0.1 | | 0.14 | | 0.1667 | | 0.0714 | |  |
| ***94*** | A_1_ | 0.2059 | − | | 0.0667 | | 0.1 | | 0.0313 | | 0.0769 | | − | | 0.0667 | | 0.0333 | | − | | − | | 0.0167 | | − | | 0.15 | | − | | − | | − | | − | | − | | 0.0476 | |  |
| ***98*** | M_5_ | − | | 0.0833 | | 0.0167 | | 0.0333 | | 0.0625 | | 0.0385 | | − | | − | | 0.05 | | 0.0385 | | − | | 0.05 | | 0.0357 | | − | | 0.125 | | 0.1364 | | 0.0333 | | 0.1 | | 0.3833 | | 0.1429 | |
| ***100*** | M_6_ | − | | − | | − | | − | | − | | − | | − | | − | | − | | **−** | | − | | − | | 0.0357 | | − | | − | | − | | − | | − | | − | | − | |
| ***102*** | M_7_ | − | | − | | 0.0167 | | − | | 0.0313 | | − | | − | | − | | 0.0333 | | − | | − | | 0.1 | | 0.0357 | | − | | 0.0208 | | 0.1364 | | 0.0667 | | 0.08 | | 0.0667 | | 0.0238 | |
| ***106*** | A_2_ | 0.0147 | | 0.5 | | 0.3333 | | 0.3333 | | 0.3438 | | 0.4808 | | 0.5 | | 0.2667 | | 0.15 | | 0.3462 | | 0.2 | | 0.0667 | | 0.1429 | | 0.2 | | 0.0833 | | 0.0455 | | 0.2 | | 0.16 | | 0.0333 | | 0.1429 | |
| ***110*** | M_8_ | − | | − | | 0.0667 | | 0.0333 | | 0.0313 | | − | | − | | 0.1333 | | 0.0833 | | − | | − | | 0.1333 | | − | | 0.05 | | 0.1875 | | 0.0909 | | 0.0667 | | 0.08 | | 0.0333 | | − | |
| ***114*** | A_3_ | 0.0588 | | 0.0833 | | 0.0667 | | 0.1667 | | 0.0625 | | 0.0577 | | − | | 0.1667 | | 0.05 | | 0.0385 | | − | | 0.15 | | − | | 0.05 | | 0.125 | | 0.0455 | | 0.0333 | | 0.06 | | 0.0667 | | 0.119 | |
| ***118*** | A_4_ | − | | − | | 0.0167 | | − | | 0.0938 | | − | | − | | − | | − | | 0.0385 | | − | | 0.0333 | | − | | − | | 0.0208 | | 0.0455 | | 0.0333 | | − | | − | | − | |
| ***122*** | M_9_ | − | | − | | − | | − | | − | | − | | − | | − | | 0.0167 | | 0.0385 | | − | | 0.05 | | − | | − | | 0.0417 | | − | | − | | 0.04 | | − | | 0.0238 | |
| ***126*** | A_5_ | 0.0441 | | − | | − | | 0.0333 | | − | | 0.0577 | | − | | − | | 0.0333 | | 0.1154 | | − | | 0.0333 | | 0.0357 | | − | | 0.0208 | | − | | − | | 0.02 | | 0.0167 | | 0.0476 | |
| ***130*** | M_10_ | − | | − | | − | | − | | − | | − | | − | | − | | − | | 0.0385 | | − | | 0.0167 | | − | | 0.1 | | − | | − | | − | | − | | 0.0167 | | 0.0238 | |
| ***134*** | A_6_ | − | | 0.0833 | | 0.1833 | | 0.0333 | | 0.0625 | | 0.0577 | | 0.25 | | 0.0333 | | 0.05 | | 0.0385 | | − | | 0.0333 | | − | | − | | − | | 0.0455 | | − | | − | | 0.05 | | 0.0238 | |
| ***138*** | A_7_ | − | | 0.0833 | | 0.1167 | | 0.1667 | | 0.0313 | | 0.1154 | | − | | 0.0333 | | 0.0333 | | − | | 0.1 | | 0.05 | | 0.0714 | | − | | 0.0208 | | 0.0909 | | − | | 0.02 | | 0.0167 | | − | |
| ***142*** | M_11_ | − | | − | | − | | − | | − | | − | | − | | 0.0333 | | − | | − | | − | | − | | 0.0714 | | 0.05 | | 0.0417 | | − | | − | | 0.02 | | − | | − | |
| ***146*** | A_8_ | 0.2059 | | 0.0833 | | 0.0333 | | 0.0333 | | 0.0625 | | 0.0577 | | − | | 0.0667 | | 0.0833 | | − | | − | | 0.0333 | | 0.0357 | | 0.1 | | 0.0833 | | − | | − | | 0.06 | | − | | − | |
| ***154*** | A_9_ | 0.0147 | | − | | 0.0333 | | − | | − | | − | | − | | 0.0333 | | − | | − | | − | | − | | − | | − | | − | | − | | 0.0333 | | − | | − | | − | |
| ***162*** | A_10_ | − | | − | | 0.0167 | | − | | − | | 0.0192 | | − | | − | | − | | − | | − | | − | | − | | − | | − | | 0.0455 | | − | | − | | − | | − | |
| ***166*** | A_11_ | − | | − | | − | | − | | − | | − | | − | | − | | 0.0167 | | − | | − | | − | | − | | − | | − | | − | | − | | − | | − | | − | |
| ***Strhae8*** |  |  | |  | |  | |  | |  | |  | |  | |  | |  | |  | |  | |  | |  | |  | |  | |  | |  | |  | |  | |  | |
| ***128*** | A'_1_ | − | | − | | − | | − | | 0.0263 | | − | | − | | − | | − | | − | | − | | − | | − | | − | | − | | − | | − | | − | | − | | − | |
| ***134*** | A'_2_ | 0.0147 | | − | | 0.25 | | 0.0667 | | 0.0263 | | 0.0769 | | − | | 0.0667 | | 0.0167 | | − | | − | | − | | − | | 0.05 | | − | | − | | − | | − | | − | | − | |
| ***138*** | A'_3_ | − | | − | | 0.1 | | 0.0333 | | 0.0526 | | 0.0769 | | − | | 0.2 | | 0.05 | | 0.0294 | | − | | 0.0667 | | 0.125 | | 0.1 | | 0.0909 | | 0.125 | | − | | 0.0192 | | 0.0167 | | 0.0682 | |
| ***139*** | M'_1_ | − | | − | | − | | − | | − | | − | | − | | − | | − | | − | | − | | − | | − | | − | | − | | − | | − | | − | | − | | 0.0227 | |
| ***142*** | A'_4_ | − | | 0.0833 | | 0.0833 | | 0.0667 | | 0.1053 | | 0.0577 | | − | | − | | 0.1 | | − | | − | | 0.0167 | | 0.05 | | − | | 0.0114 | | 0.025 | | 0.1 | | 0.0385 | | 0.0167 | | 0.0682 | |
| ***146*** | A'_5_ | 0.3382 | | 0.3333 | | 0.1333 | | 0.3667 | | 0.3158 | | 0.3269 | | − | | 0.3333 | | 0.2167 | | 0.1176 | | 0.6 | | 0.25 | | 0.15 | | 0.1 | | 0.2273 | | 0.35 | | 0.1 | | 0.1923 | | 0.1667 | | 0.1136 | |
| ***150*** | A'_6_ | 0.0147 | | 0.0833 | | 0.0667 | | 0.0333 | | 0.0263 | | − | | − | | 0.1 | | 0.0333 | | 0.1765 | | − | | 0.0833 | | 0.075 | | 0.05 | | 0.0795 | | 0.05 | | 0.1 | | 0.1154 | | 0.0667 | | 0.0455 | |
| ***154*** | A'_7_ | 0.0294 | | − | | 0.0167 | | − | | − | | 0.0577 | | − | | − | | 0.0167 | | 0.0588 | | − | | 0.0167 | | 0.025 | | 0.05 | | − | | 0.025 | | 0.0333 | | − | | − | | 0.0455 | |
| ***158*** | A'_8_ | 0.0294 | | 0.1667 | | 0.1167 | | 0.1667 | | 0.1316 | | 0.2115 | | 0.5 | | 0.0333 | | 0.05 | | − | | − | | − | | 0.025 | | 0.05 | | − | | 0.025 | | − | | − | | − | | − | |
| ***162*** | A'_9_ | − | | − | | 0.0167 | | − | | 0.0526 | | 0.1346 | | 0.25 | | − | | 0.0333 | | − | | − | | − | | 0.025 | | − | | 0.0114 | | − | | − | | − | | 0.0333 | | − | |
| ***166*** | A'_10_ | 0.0735 | | 0.1667 | | 0.1 | | 0.1 | | − | | − | | 0.25 | | 0.1333 | | 0.05 | | 0.2353 | | − | | 0.0667 | | 0.05 | | 0.1 | | 0.0455 | | 0.075 | | 0.1 | | 0.1154 | | 0.0167 | | 0.0682 | |
| ***170*** | M'_2_ | − | | − | | 0.0333 | | − | | − | | − | | − | | 0.1 | | 0.05 | | 0.0588 | | 0.1 | | 0.2 | | 0.1 | | 0.05 | | 0.1023 | | 0.05 | | 0.1 | | 0.0962 | | 0.1 | | 0.1364 | |
| ***174*** | M'_3_ | 0.4118 | | − | | 0.0333 | | 0.0333 | | 0.0263 | | 0.0192 | | − | | − | | 0.1 | | 0.0588 | | 0.1 | | 0.1167 | | 0.175 | | 0.15 | | 0.2159 | | 0.125 | | 0.1667 | | 0.1538 | | 0.1 | | 0.1591 | |
| ***178*** | M'_4_ | − | | − | | − | | − | | 0.0263 | | − | | − | | − | | 0.0333 | | − | | − | | − | | 0.025 | | 0.05 | | 0.0455 | | − | | 0.0667 | | 0.0962 | | 0.0667 | | 0.0682 | |
| ***182*** | M'_5_ | − | | − | | 0.0167 | | 0.0333 | | 0.1053 | | − | | − | | 0.0333 | | 0.15 | | 0.1176 | | 0.2 | | 0.1167 | | 0.1 | | 0.15 | | 0.1136 | | 0.125 | | 0.1 | | 0.1538 | | 0.3167 | | 0.1818 | |
| ***186*** | M'_6_ | − | | − | | − | | − | | 0.0526 | | − | | − | | − | | 0.0167 | | 0.1176 | | − | | 0.0667 | | 0.05 | | 0.1 | | 0.0568 | | 0.025 | | 0.1333 | | 0.0192 | | 0.1 | | 0.0227 | |
| ***195*** | A'_11_ | − | | − | | − | | − | | − | | − | | − | | − | | 0.0333 | | − | | − | | − | | − | | − | | − | | − | | − | | − | | − | | − | |
| ***198*** | A'_12_ | 0.0882 | | 0.1667 | | 0.0333 | | 0.1 | | 0.0263 | | 0.0385 | | − | | − | | 0.0333 | | 0.0294 | | − | | − | | 0.025 | | − | | − | | − | | − | | − | | − | | − | |
| ***208*** | A'_13_ | − | | − | | − | | − | | 0.0263 | | − | | − | | − | | 0.0167 | | − | | − | | − | | − | | − | | − | | − | | − | | − | | − | | − | |
| ***Strhae9*** |  |  | |  | |  | |  | |  | |  | |  | |  | |  | |  | |  | |  | |  | |  | |  | |  | |  | |  | |  | |  | |
| ***130*** | M"_1_ | − | | − | | − | | − | | − | | − | | − | | − | | − | | − | | − | | 0.0167 | | − | | − | | − | | − | | − | | − | | − | | − | |
| ***138*** | A"_1_ | 0.0455 | | − | | − | | 0.1 | | 0.0455 | | 0.025 | | − | | − | | 0.0192 | | − | | − | | − | | 0.0294 | | − | | − | | 0.0357 | | − | | − | | − | | 0.0556 | |
| ***141*** | A"_2_ | − | | 0.5 | | 0.1429 | | 0.2 | | 0.2273 | | 0.125 | | − | | 0.0417 | | 0.0192 | | − | | − | | − | | − | | − | | − | | − | | − | | − | | − | | − | |
| ***144*** | A"_3_ | − | | − | | 0.0714 | | − | | − | | 0.025 | | − | | 0.0417 | | 0.0192 | | 0.0667 | | 0.1 | | 0.0167 | | 0.1471 | | 0.0556 | | 0.0444 | | − | | − | | 0.06 | | 0.0333 | | 0.0833 | |
| ***148*** | A"_4_ | 0.1136 | | 0.3 | | 0.1786 | | 0.2333 | | 0.0455 | | 0.05 | | − | | 0.2083 | | 0.0577 | | 0.0333 | | − | | 0.1 | | 0.0588 | | 0.0556 | | 0.1111 | | 0.1429 | | 0.1667 | | 0.02 | | 0.05 | | 0.1389 | |
| ***152*** | M"_2_ | 0.0227 | | − | | 0.1429 | | 0.0333 | | − | | 0.2 | | − | | 0.0417 | | 0.0192 | | 0.1333 | | − | | 0.0333 | | 0.1765 | | 0.2778 | | 0.1111 | | 0.1071 | | 0.2 | | 0.14 | | 0.15 | | 0.0556 | |
| ***155*** | A"_4_ | − | | − | | − | | − | | − | | − | | − | | − | | 0.0192 | | − | | − | | − | | − | | − | | − | | − | | − | | − | | − | | − | |
| ***156*** | M"_3_ | 0.1364 | | − | | − | | 0.0333 | | 0.1818 | | − | | − | | − | | 0.2885 | | 0.0667 | | 0.2 | | 0.1667 | | 0.0882 | | 0.0556 | | 0.1333 | | 0.1786 | | 0.0333 | | 0.2 | | 0.0833 | | 0.1944 | |
| ***160*** | M"_4_ | 0.0682 | | 0.1 | | 0.0714 | | 0.0333 | | 0.0455 | | 0.05 | | − | | 0.1667 | | 0.1154 | | 0.1333 | | 0.1 | | 0.2333 | | 0.1471 | | 0.1667 | | 0.1333 | | 0.0357 | | 0.2 | | 0.12 | | 0.1333 | | 0.0833 | |
| ***162*** | A"_5_ | − | | − | | − | | − | | 0.1364 | | − | | − | | − | | − | | − | | − | | − | | − | | − | | − | | − | | − | | − | | − | | − | |
| ***164*** | A"_6_ | 0.25 | | − | | 0.0357 | | 0.1333 | | 0.0455 | | 0.275 | | − | | 0.125 | | 0.1346 | | 0.1667 | | 0.2 | | 0.05 | | 0.0294 | | 0.2222 | | 0.1 | | 0.1071 | | − | | 0.06 | | 0.1333 | | 0.0556 | |
| ***165*** | A"_7_ | − | | − | | − | | − | | − | | − | | − | | − | | 0.0192 | | − | | − | | − | | − | | − | | − | | − | | − | | − | | − | | − | |
| ***168*** | A"_8_ | 0.2727 | | − | | 0.2143 | | 0.0667 | | − | | 0.2 | | 1 | | 0.1667 | | 0.1923 | | 0.1 | | 0.1 | | 0.1167 | | 0.0882 | | 0.0556 | | 0.1 | | 0.1786 | | − | | 0.1 | | 0.15 | | 0.1389 | |
| ***172*** | M"_5_ | 0.0455 | | − | | − | | − | | 0.0909 | | − | | − | | − | | 0.0962 | | 0.1333 | | 0.1 | | 0.0667 | | 0.1176 | | 0.0556 | | 0.1 | | 0.1071 | | 0.1 | | 0.06 | | 0.1333 | | − | |
| ***176*** | M"_6_ | 0.0227 | | 0.1 | | − | | 0.0333 | | 0.0909 | | 0.05 | | − | | 0.0833 | | − | | 0.0667 | | 0.1 | | 0.1 | | 0.0588 | | − | | 0.0778 | | 0.0357 | | 0.1333 | | 0.1 | | 0.0667 | | 0.0556 | |
| ***180*** | M"_7_ | − | | − | | − | | 0.0667 | | 0.0909 | | − | | − | | 0.0417 | | − | | 0.0333 | | 0.1 | | 0.0333 | | 0.0588 | | 0.0556 | | 0.0333 | | − | | 0.0333 | | 0.06 | | 0.05 | | 0.0556 | |
| ***184*** | M"_8_ | − | | − | | 0.1429 | | − | | − | | − | | − | | 0.0417 | | − | | 0.0667 | | − | | 0.0333 | | − | | − | | 0.0333 | | 0.0714 | | 0.0333 | | 0.02 | | 0.0167 | | 0.0278 | |
| ***188*** | M"_9_ | 0.0227 | | − | | − | | 0.0667 | | − | | − | | − | | − | | − | | − | | − | | 0.0333 | | − | | − | | 0.0222 | | − | | 0.1 | | − | | − | | 0.0556 | |
| ***192*** | M"_10_ | − | | − | | − | | − | | − | | − | | − | | 0.0417 | | − | | − | | − | | − | | − | | − | | − | | − | | − | | 0.02 | | − | | − | |
| ***200*** | M"_11_ | − | | − | | − | | − | | − | | − | | − | | − | | − | | − | | − | | − | | − | | − | | − | | − | | − | | 0.02 | | − | | − | |
| ***206*** | M"_12_ | − | | − | | − | | − | | − | | − | | − | | − | | − | | − | | − | | − | | − | | − | | − | | − | | − | | 0.02 | | − | | − | |

**Table S5.** Pairwise *FST* values (below diagonal) and pairwise *Jost’s D* values (above diagonal) for *Strhae3*, *Strhae8* and *Strhae9*.

|  | **AB** | **WG** | **AOF** | **HZA** | **HZB** | **GL** | **PRAM** | **STS** |
| --- | --- | --- | --- | --- | --- | --- | --- | --- |
| **AB** | - | 0.3620 | 0.2853 | 0.1445 | 0.1416 | 0.2508 | 0.2708 | 0.2978 |
| **WG** | 0.0631 | - | 0.2221 | 0.0401 | 0.2048 | 0.0691 | 0.2829 | 0.2923 |
| **AOF** | 0.0460 | 0.0238 | - | 0.0000 | 0.0056 | 0.4157 | 0.0137 | 0.0275 |
| **HZA** | 0.0256 | 0.0057 | -0.0005 | - | 0.0521 | 0.1885 | 0.0116 | 0.1138 |
| **HZB** | 0.0344 | 0.0347 | 0.0014 | 0.0061 | - | 0.3488 | 0.0057 | 0.0436 |
| **GL** | 0.0786 | 0.0164 | 0.0609 | 0.0293 | 0.0724 | - | 0.5149 | 0.4242 |
| **PRAM** | 0.0506 | 0.0370 | 0.0006 | 0.0048 | 0.0064 | 0.0761 | - | 0.0254 |
| **STS** | 0.0420 | 0.0331 | 0.0028 | 0.0104 | 0.0085 | 0.0652 | 0.0055 | - |
